# Supplementary material for: The Strong Anti-Tumor Effect of Smp24 in Lung Adenocarcinoma A549 Cells Depends on Its Induction of Mitochondrial Dysfunctions and ROS Accumulation
Source: Toxins (Basel). 2022 Aug 27;14(9):590. doi: 10.3390/toxins14090590 (PMC9502404; doi:10.3390/toxins14090590)
Supplement: Supplementary file 1 [file toxins-14-00590-s001.zip › toxins-1861174-supplementary.pdf]

toxins

MDPI

The strong anti-tumor effect of Smp24 in lung Adenocarcinoma A549 Cells depends on its inductions of mitochondrial dysfunctions and ROS accumulation

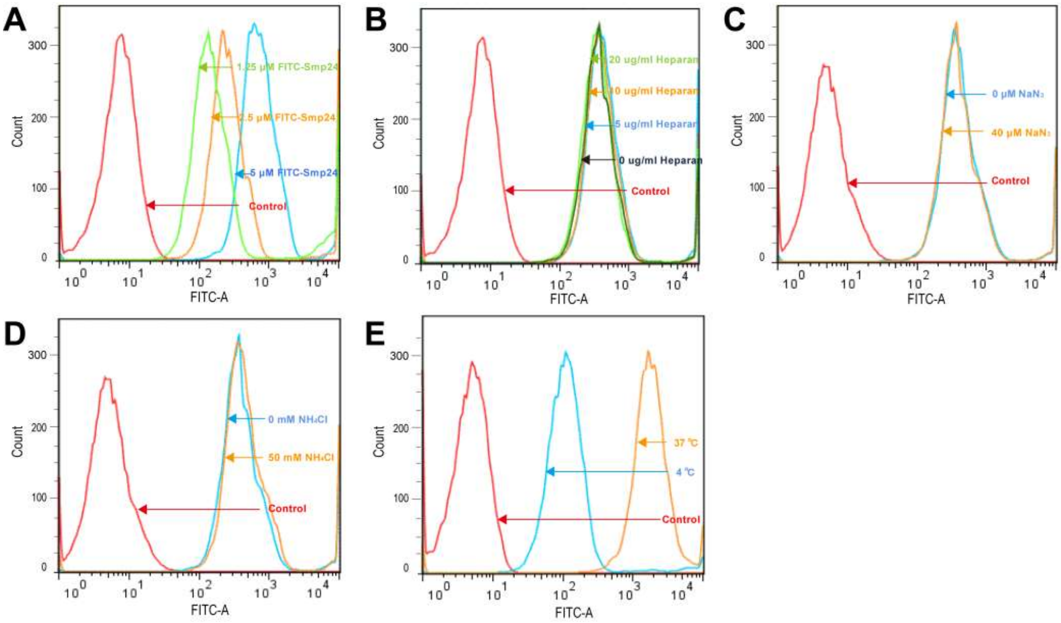

Figure S1. Internalization of Smp24 into A549 cells. (A) The internalization of FITC-labeled Smp24 into A549 cells after 6 h of treatment. (B-E) Effect of heparan sulfate, NaN_3, NH_4Cl and temperature on the intracellular uptake of FITC-labeled Smp24 for 6 h, respectively. The values are presented as mean ± SEM (n = 3).
